# Development of SimCells as a novel chassis for functional biosensors

**DOI:** 10.1038/s41598-017-07391-6

**Published:** 2017-08-03

**Authors:** Cordelia P. N. Rampley, Paul A. Davison, Pu Qian, Gail M. Preston, C. Neil Hunter, Ian P. Thompson, Ling Juan Wu, Wei E. Huang

**Affiliations:** 1 Department of Engineering Science, University of Oxford, Parks Road, Oxford, OX1 3PJ United Kingdom; 2 Department of Molecular Biology and Biotechnology, University of Sheffield, Sheffield, S10 2TN United Kingdom; 3 Department of Plant Sciences, University of Oxford, South Parks Road, Oxford OX1 3RB, Oxford, United Kingdom; 4 The Centre for Bacterial Cell Biology, Institute for Cell and Molecular Biosciences, Newcastle University, Richardson Road, Newcastle upon Tyne, NE2 4AX United Kingdom

## Abstract

This work serves as a proof-of-concept for bacterially derived SimCells (Simple Cells), which contain the cell machinery from bacteria and designed DNA (or potentially a simplified genome) to instruct the cell to carry out novel, specific tasks. SimCells represent a reprogrammable chassis without a native chromosome, which can host designed DNA to perform defined functions. In this paper, the use of *Escherichia coli* MC1000 ∆*min*D minicells as a non-reproducing chassis for SimCells was explored, as demonstrated by their ability to act as sensitive biosensors for small molecules. Highly purified minicells derived from *E. coli* strains containing gene circuits for biosensing were able to transduce the input signals from several small molecules (glucarate, acrylate and arabinose) into the production of green fluorescent protein (GFP). A mathematical model was developed to fit the experimental data for induction of gene expression in SimCells. The intracellular ATP level was shown to be important for SimCell function. A purification and storage protocol was developed to prepare SimCells which could retain their functions for an extended period of time. This study demonstrates that SimCells are able to perform as ‘smart bioparticles’ controlled by designed gene circuits.

## Introduction

Synthetic biology aims to apply engineering principles to make reliable, robust and predictable biological systems. It offers enormous potential to address global concerns within the energy, health and water industries, with applications in technologies such as engineered sensing systems^1^, biofuel synthesis^2^ and medical diagnostics and treatments^3, 4^. Unpredictable biological performance and issues surrounding the use of GMOs are among the major barriers to realising the full potential of synthetic biology^5, 6^. Biological complexity makes standardised bioparts less robust and predictable, and insufficiently reliable for use in engineered processes^6^. Such complexity is largely caused by inherent stochastic gene expression^7^ and the interactions between a host’s native gene network and designed circuits. In addition, poor public perception of GMOs may hamper the broad application of synthetic cells^8^.

To address the challenges of complexity and the issue of GMOs, we propose a new chassis concept for synthetic biology – SimCells (Simple cells). SimCells inherit the shell, or ‘hardware’ from parent cells, which can be optimized with specific cellular properties by gene modification of the parent cells. SimCells have no chromosome, and thus, no ability to reproduce. They instead harbour engineered and designed DNA as ‘software’, which encodes pre-defined functions, whilst having many of the ‘background’ gene networks of native cells eliminated. It has been demonstrated that simple gene circuits in cell-free systems without complex background interference would be more predictable, as shown by the commercial PURExpress kit and in reports on the expression of gene circuits in cell free systems^9, 10^. SimCells are able to perform basic functions as some mRNA, rRNA and tRNA remain in the cellular ‘shell’ even in the absence of the chromosome. Therefore, SimCells could be seen as designable ‘smart bioparticles’ that avoid the controversial label of being a GMO. They are a new type of chassis, falling between a living cell and a cell-free system. They have the potential to be stable and robust as they eliminate unnecessary gene networks in native organisms, but maintain the cell machinery required for faithful gene expression^11^.

There are several ways to make chromosome-free cells^12–14^ and the designed DNA can be introduced into parent SimCells as plasmids. One possible source of SimCell ‘hardware’ is minicells that are produced by some rod-shaped bacteria. MinD plays an important role in bacterial division along with MinC and MinE, by localising the *E. coli* divisional machinery to the mid-cell in preference to other sites. A mutation in the *min*D gene results in incorrect placement of the septum, and hence minicell formation^15^. Minicells are small, chromosome-free cells formed as a result of aberrant cell division, where cells divide near the pole ends as opposed to the mid-cell region^16, 17^. The chromosome-free minicells contain ribosomes, tRNA and structural components, and plasmids can be inherited from the parent cells^18–20^. Plasmids are preferentially located at the poles and enter the minicells via an active partition system or by random distribution^21^. Minicells from both Gram positive and negative bacterial species have been reported^22^. They could have evolved as a survival mechanism^23^ as their functions include acting as vehicles for the removal of toxic and antimicrobial compounds from the cell, inter-cell signalling, motility and predation within biofilms^19^. They have been utilised to study a variety of mechanisms including molecular transport, protein synthesis and cell division^24–27^. In particular, they were used for RNA and protein synthesis studies during the 1970s due to their absence of cross-talk and interference between signal transduction pathways^28^. They have been demonstrated to transcribe and translate plasmid encoded DNA^29, 30^. Hence, minicells could offer a simple chassis for the exploration of *de novo* synthesised genes and minimised gene sets.

In this paper, *E. coli* MC1000 ∆*min*D was used as the parent cell strain for the production of minicell-based SimCells^15^. This work examines the ability of these minicells to produce plasmid-encoded GFP in response to three small inducer molecules: acrylate, L-arabinose and glucarate, using tightly regulated and robust systems. This system has the potential to be utilised in other bacterial species and genetic elements to simplify and facilitate expression of engineered gene circuits.

## Results

### Minicells can be continuously produced from parent cells

**Figure 1:**
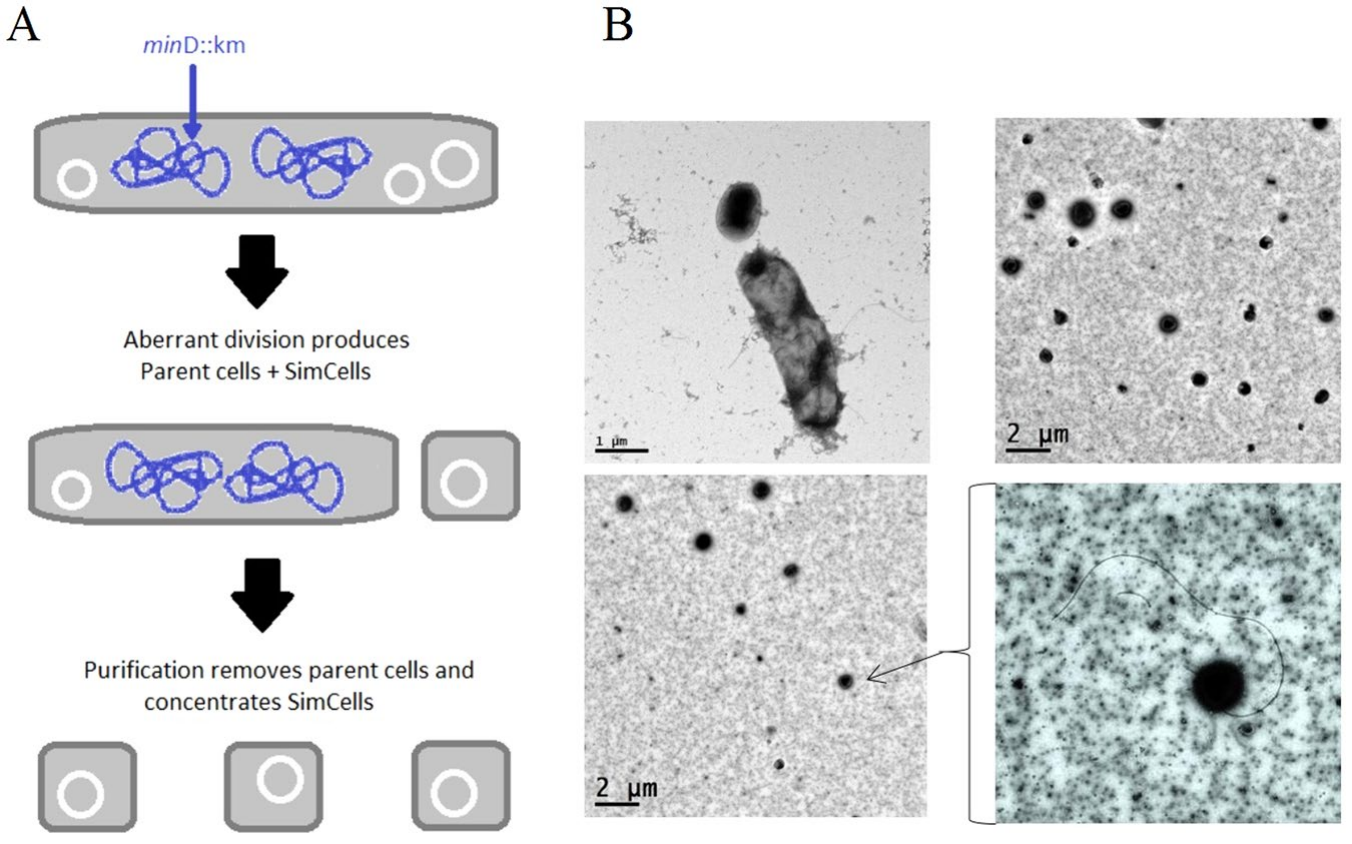
(**A**) Schematic showing how *E. coli* cells with a *min*D gene knockout are able to produce SimCells without a chromosome, but with plasmids; (**B**) Electron micrographs showing *E. coli* MC1000 *min*D::km producing SimCells of different sizes and morphologies, including flagella.

A schematic of SimCells produced from *E. coli* MC1000 ∆*min*D is shown in Fig. 1A. SimCells pinch and bud off from the outer membrane of the parent cell, forming detached near-spherical miniature cells with approximate diameters of 620 ± 222 nm based on the TEM images shown in Fig. 1B. It was also noted in the TEM images that many minicells had flagella (Fig. 1), and light microscopy revealed that cells were motile for several hours after purification. Parent cells can continuously produce chromosome-free SimCells via a ‘budding’-like process (Fig. S1A). The size difference between the parent cell and SimCells is highlighted in Fig. S1B and the high uniformity of the purified SimCells can be seen in Fig. S1C.

### Parent cell induction

**Figure 2:**
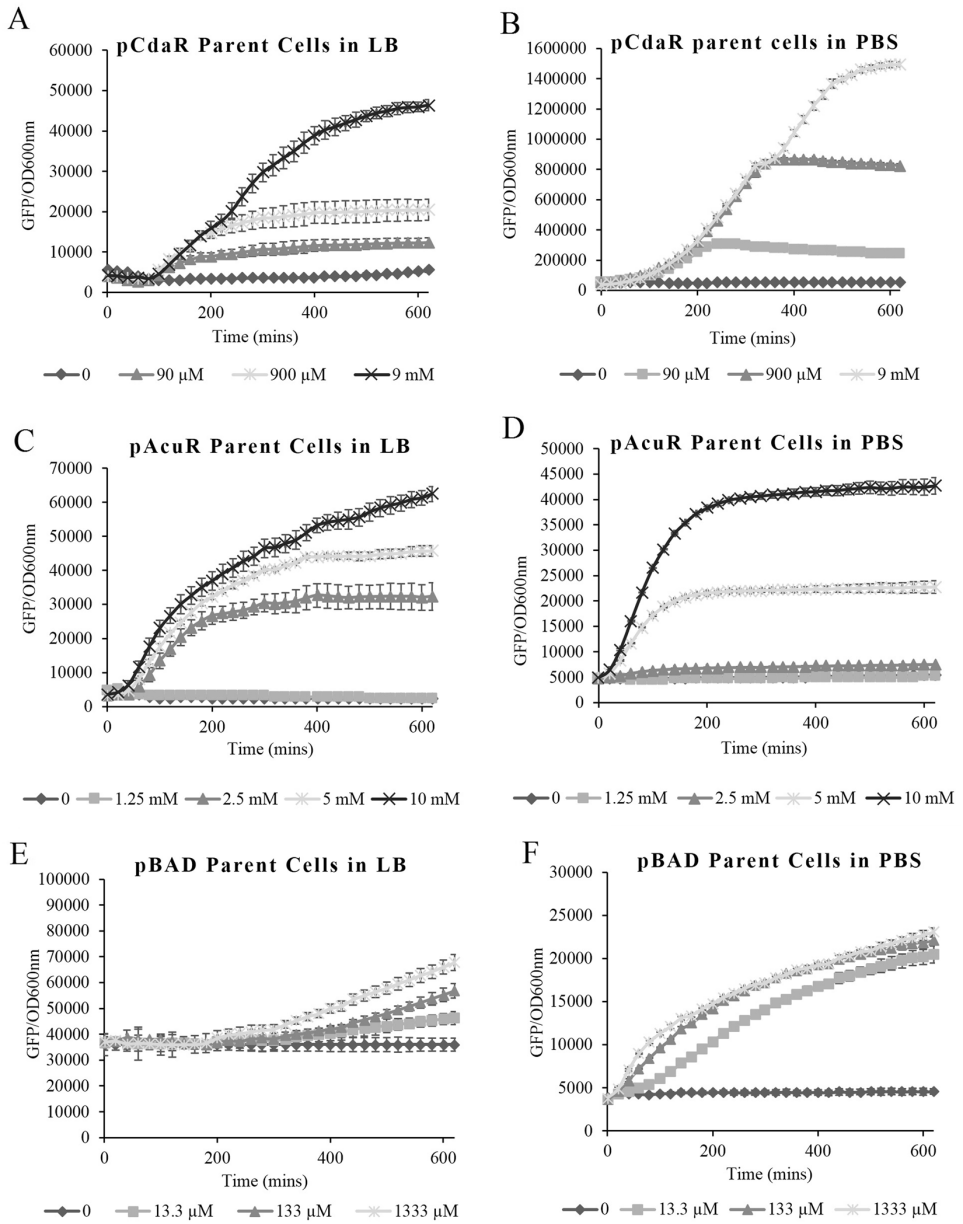
Induction of parent cells in LB and PBS over time, as determined by GFP fluorescence per unit OD at 600 nm (n = 4). Error bars denote one standard deviation above and below the mean. pCdaR parent cells induced by glucarate in LB (**A**) and PBS (**B**), pAcuR parent cells induced by acrylate in LB (**C**) and PBS (**D**), and pBAD parent cells induced by arabinose in LB (**E**) and PBS (**F**). The OD 600 nm curves for the parent cells induced in PBS are shown in Fig. S3.

Three different regulation systems were chosen in this study, and the plasmid maps are shown in Fig. S2: pCdaR contains a positive autoregulation system in which the activator CdaR is regulated by its own promoter, induced by CdaR binding glucarate^31^; pAcuR contains a negative autoregulation system in which AcuR is a repressor, acting in the absence of acrylate, regulated by its own promoter^31, 32^; pBAD contains a dual regulation system controlled by an activator AraC, and a global repressor cyclic AMP receptor protein (CRP)^33^. Induction of parent cell cultures containing these regulation systems was tested to establish sensitivity, prior to SimCell purification (Fig. 2). In all three cases, cells with pCdaR, pAcuR and pBAD could be induced by different concentrations of glucarate, acrylate and arabinose, respectively, in both LB and PBS buffer. In the case of induction in LB medium, parent cells with pAcuR and pBAD showed negligible leaky expression, whereas cells with pCdaR had a leaky background. In contrast, when these cells were induced in PBS buffer, all controls (no inducers) exhibited no increase in fluorescence resulting from GFP production after 10 h, demonstrating a tightly regulated system with low leaky expression (Fig. 2). Although *E. coli* was able to grow using acrylate and L-arabinose as sole carbon sources^33^, *E. coli* MC1000 ∆*min*D carrying pAcuR or pBAD showed no growth in PBS buffer (Fig. S3). However, acrylate and arabinose were still able to activate GFP expression, suggesting that gene induction could occur without cell growth (Fig. S3). *E. coli* can grow using glucarate as a sole carbon source^34^; *E. coli* MC1000 ∆*min*D carrying pCdaR showed obvious growth in PBS when glucarate concentrations were higher than 900 µM (Fig. S3). In all these regulation systems, cells induced by the inducing agent showed a significant increase in GFP production (Fig. 2), demonstrating that these systems were suitable for testing in the SimCell chassis.

### Purified SimCells are able to respond to inducers

Removal of chromosome-containing parent cells yielded a highly pure SimCell suspension of fewer than ~70 parent cells/ml (from a culture of ~4 × 10^9^ parent cells/ml) (Table 1 and Fig. S1C). Figure S4 shows that a cell population greater than 7 × 10^6^ parent cells/ml is required to have a detectable GFP expression (as measured in a microplate reader) in cells with pCdaR induced by various concentrations of glucarate. This suggests that the 70 parent cells/ml remaining in the purified SimCells do not contribute to any GFP measurement. Hence, in the following induction experiments, it was the SimCells that contributed to GFP expression rather than the very few parent cells remaining in the SimCell suspensions.

**Table 1:** Summary of induction ratios in parent cells and purified SimCells and plate counts of SimCells.

	Maximum Parent cell fold induction	Maximum SimCell fold induction	Viable cells after purification CFU/ml
pAcuR	8.86 ± 0.19	5.56 ± 0.14	63 ± 12
pCdaR	34.94 ± 0.42	3.07 ± 0.06	0 ± 0
pBAD	12.46 ± 0.04	2.42 ± 0.04	73 ± 15

**Figure 3:**
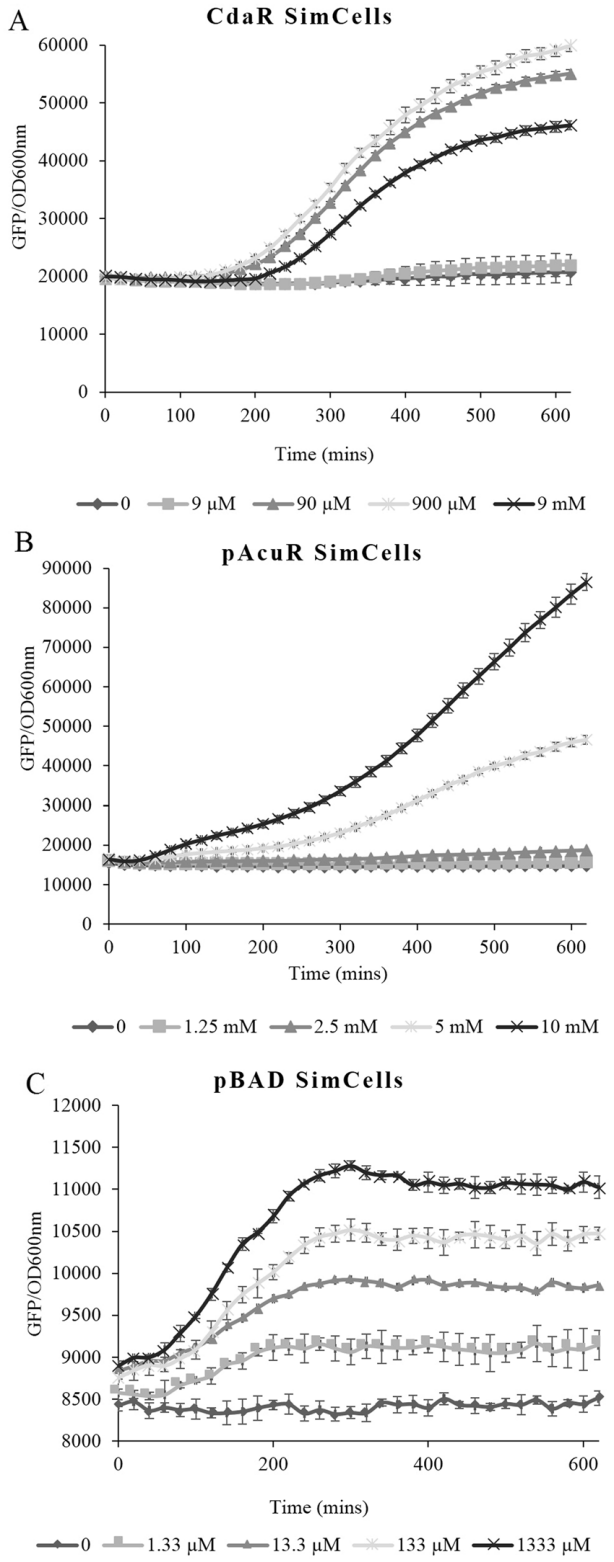
Induction of purified SimCells in PBS over time, as determined by GFP fluorescence per unit OD at 600 nm (n = 4). Error bars denote one standard deviation above and below the mean. (**A**) pCdaR SimCells induced by glucarate (significant induction relative to the control (p < 0.05) at 90 µM glucarate and above). (**B**) pAcuR SimCells induced by acrylate (significant induction at 5 mM acrylate and above). (**C**) pBAD SimCells induced by arabinose (significant induction at all concentrations tested). The OD 600 nm curves are shown in Fig. S5.

Purified SimCells were subsequently induced with varying concentrations of glucarate, acrylate and L-arabinose, as shown in Fig. 3. GFP expression increased in a dose-dependent response to the inducing agents, whilst GFP in the ‘no inducer’ controls showed no increase during the 10 hr induction period. The optical densities of SimCells in all cases, including SimCells with high concentrations of glucarate, showed no change (Fig. S5), suggesting that the SimCells are highly purified and show no growth of parent cells. SimCells derived from non-dividing minicells can therefore function as biosensors by responding to several inducers and producing a detectible signal in the form of protein.

### Mathematical Model to simulate the performance of SimCells with pBAD

**Figure 4:**
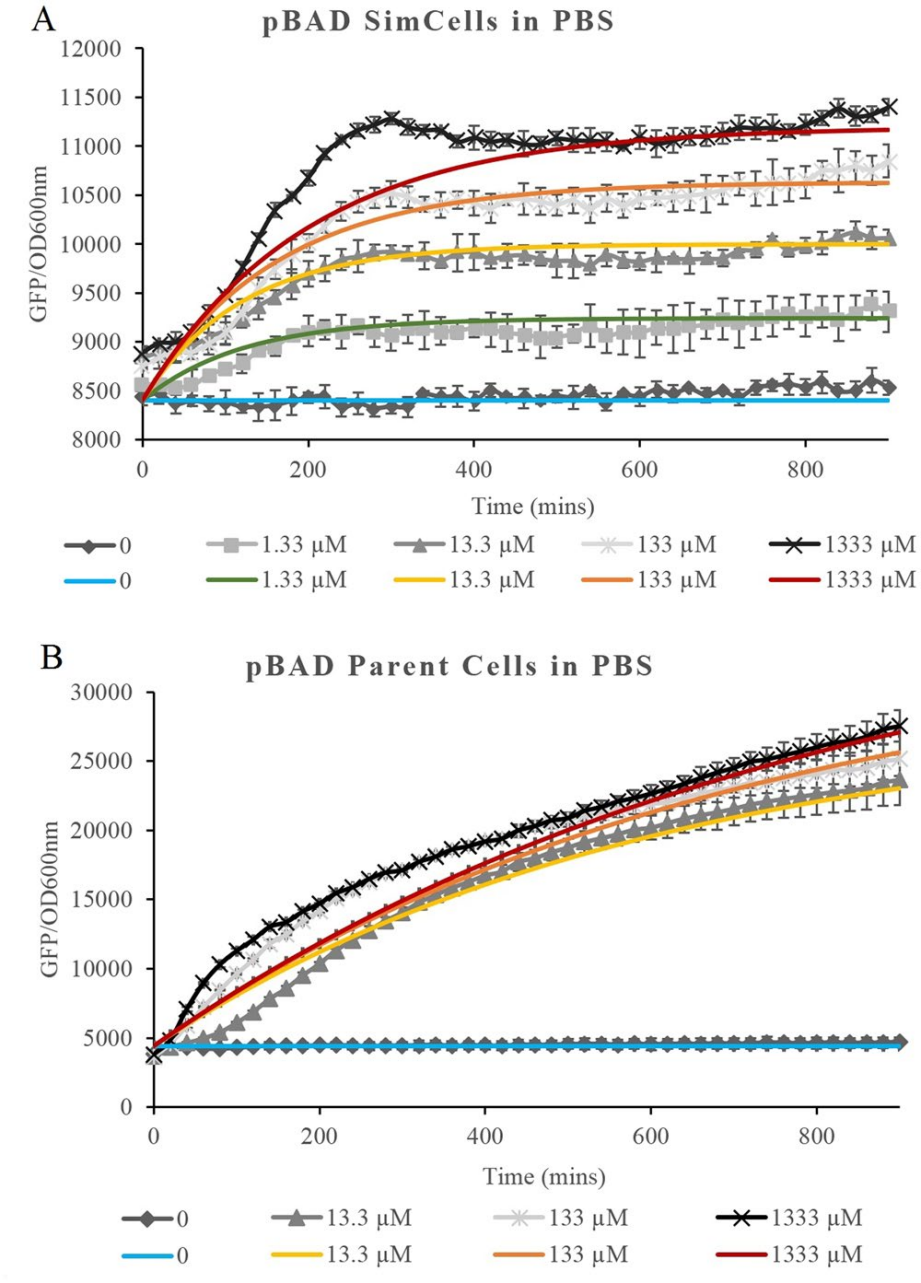
Experimental (black and white) & modelled (coloured) data of the induction over time as determined by GFP fluorescence per unit OD at 600 nm of (**A**) pBAD SimCells in PBS with L-arabinose and (**B**) pBAD Parent Cells in PBS with L-arabinose.

In this study, we developed a modified mathematical model based on Uri Alon’s work^35^ to simulate the performance of SimCells in order to identify their key limits and help improve SimCell-based design (see supplementary information). As arabinose induction in *E. coli* by *ara*C and its promoter has been studied intensively^36^, the parameters related to this regulation have been well documented^36–38^. We applied the model to simulate the performance of SimCells with the pBAD system (Fig. 4 and Table S1). Since SimCells are a simple system without a continuous energy supply, it is reasonable to introduce a decay term α_d_·P in the model which curbs protein (GFP) production (supplementary information, Equation 9). In the pBAD system, the expression of *ara*C, controlled by a constitutive promoter, was assumed to be constant. Using the intrinsic parameters reported in the literature (Table S1), the model fitted the experimental data for both SimCells and parent cells in PBS (Fig. 4). With the exception of *α*_d_, the decay constant of protein production, and the fact that the initial background may vary in different cases, all other intrinsic parameters, such as the properties of protein-promoter and protein-molecule interactions, were the same to fit all experimental data. Since parent cells contain a mixture of SimCells and normal growing cells, that can use arabinose as an energy source, the *α*_d_ (1.25–1.67 × 10^−3^ min^−1^) of parent cells in PBS is smaller than the *α*_d_ (5–8.33 × 10^−3^ min^−1^) of SimCells (Table S1). For higher concentrations of arabinose, the slightly smaller value of *α*_d_ was applied.

When the experimental data was analysed, it was shown that arabinose induction of pBAD-containing SimCells and parent cells in PBS (Figs 2F and 3C respectively) was activated rapidly, whilst the induction of parent cells in LB was delayed by at least 160 min (Fig. S6). One possibility drawn from the information provided by the model is that as the cells grow and divide rapidly over the first 160 minutes following induction, the activated regulatory protein AraC has insufficient time to reach the threshold concentration required for activating *gfp* expression before the next cell division takes place, and hence no induction of GFP expression was observed. When cell growth and division slowed down after 160 min, the cells were able to accumulate a sufficient amount of activated AraC to switch on GFP expression (Fig. S6), whereas without division, AraC in parent cells or SimCells at PBS reached the threshold earlier to allow expression of GFP. Further research will be required to confirm this accumulation effect in non-dividing cells.

### ATP and temperature affect SimCell performance

**Figure 5:**
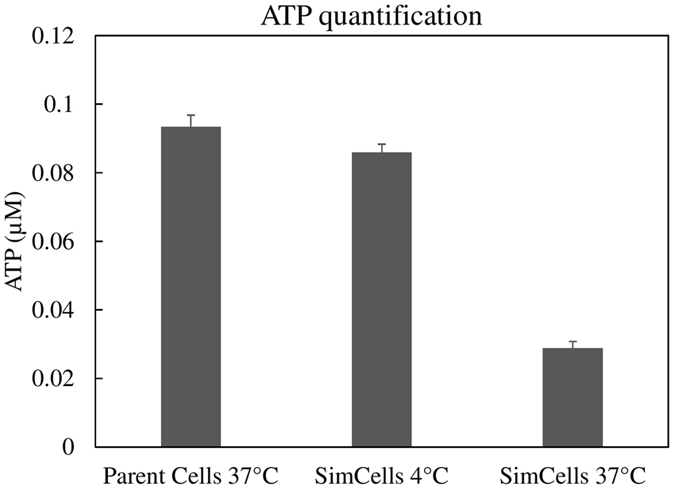
Total ATP from 100 µL SimCells with pCdaR (OD600 = ~0.1) using a luciferase based assay kit, BacTiterGlo. Samples included: non-purified parent cells maintained at 37 °Cand purified SimCells maintained for 24 h at 4 °C and at 37 °C, to demonstrate the temperature dependency of the ATP concentration. Error bars represent one standard deviation above and below the mean (n = 3).

The temperature during the purification and storage steps greatly affected the overall ability of the SimCells to act as biosensors. It was observed that SimCells purified and stored at 4 °C had significantly higher induction than those prepared and stored at 37 °C, when exposed to the inducing agent. It is hypothesised that SimCells purified and stored at 37 °C will have a lower intracellular ATP level, which is critical to drive the SimCell response. This was confirmed using the BacTiterGlo ATP assay in Fig. 5, which shows that the 4 °C maintained SimCells contained approximately 3 times more ATP than control cells maintained at 37 °C for 24 hours. These findings led to the modification of the purification protocol to incorporate a 4 °C incubation step that increased the functionality of the SimCells. Furthermore, SimCells remained functional for prolonged periods in storage, as SimCells suspended in PBS that were tested after being maintained at 4 °C for 200 days were still inducible (Fig. S7).

### Microscopic examination of SimCells in response to inducers

**Figure 6:**
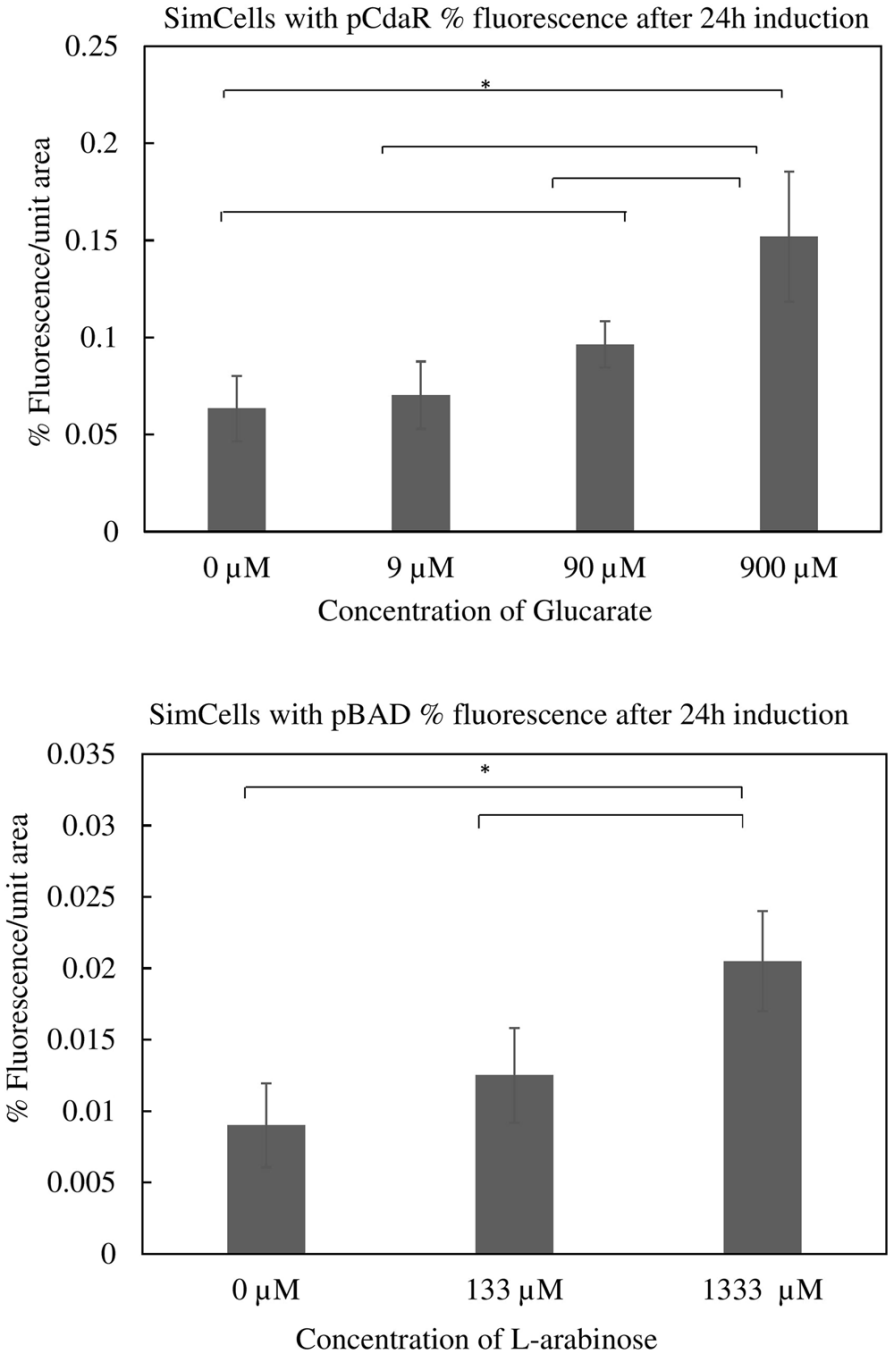
Quantification of fluorescent cells of SimCells with (**A**) pCdaR and (**B**) pBAD from light microscope images, expressed as % fluorescence per unit area. The data displayed are the average of 4 images, with one standard deviation above and below the mean. Asterisks denote significance at a 95% confidence level.

Images of post-induction SimCells in a 96 well plate were captured using light microscopy. Although the uninduced SimCell control contained several fluorescent SimCells, more SimCells became fluorescent as the concentration of the inducers increased. This was quantified and analysed using ImageJ (Fig. 6). The imaging analysis of SimCells containing pCdaR shows a significant difference between the control and those with 90 µM (p < 0.01) or 900 µM (p < 0.001) glucarate (Fig. 6A). A difference seen between the control and 9 µM glucarate (p = 0.40) was not significant, and was consistent with the result observed when using the microplate reader as shown in Fig. 3A. The imaging analysis of SimCells containing pBAD shows that there was a significant difference between the control and 1333 µM arabinose (p < 0.01) (Fig. 6B).

In order to enumerate any parent cells or contaminants, purified SimCells suspended in PBS were plated out on LB agar, and the CFU/ml of each sample was counted. The results are summarised in Table 1, which indicates that negligible parent cells remained within purified SimCells. This is consistent with the optical density (OD 600 nm), which was monitored throughout all fluorescence experiments, which demonstrated that there was no detectable increase over the duration tested. In all cases, the extent of induction (maximum fluorescence values divided by the uninduced control fluorescence) was lower for SimCells than parent cells.

## Discussion

Biosensors are frequently used to selectively and sensitively detect key molecules in medical, forensic and environmental fields^39^. In the future, synthetic biology should have a large role in producing useful biosensors, particularly within challenging environments such as contaminated soil and water, or within the human body for tumour detection^40^.

There are many potential applications of bacterial biosensors in agriculture, energy production and storage, and medicine. However, the cell’s natural tendency to replicate and transfer DNA poses many concerns with regard to the release of GMOs. Consequently, whole-cell genetically engineered biosensors need to be safely confined and disposed of using appropriate guidelines, which limits their usage both medically and environmentally. Besides their inability to grow and replicate, SimCells can be designed to prevent possible dissemination of synthetic genetic material (e.g. plasmids in SimCells) into the environment. For example, DNA design in SimCells can be built on non-conjugative plasmid backbones (e.g. those without *oriT* and *tra* genes)^41, 42^; and pili-free bacteria can be chosen as the parents of SimCells to prevent possible horizontal gene transfer^43^. Recent technology has also produced bacteria specifically designed to require synthetic amino acids for growth, thus limiting their proliferation in the case of their accidental release into the environment^44, 45^. Other technologies have developed cell-free biosensing systems utilising enzymes fixed in two dimensions^10^. While this offers a rapid, user-friendly and low-cost method of biosensing, using a cell-based system may enable higher sensitivity, as compartmentalisation may allow lower concentrations of molecules to be detected. In a 1 µm-size bacterial cell, one molecule per cell equates to around 1 nM concentration^35^, whereas in a minicell the volume is around 5 to 10 times smaller, leading to a higher effective intracellular concentration, enabling a potentially more sensitive method of small metabolite detection. For example, in this study the detection limit of arabinose in pBAD SimCells was 1.33 µM (Fig. 3) compared to a value in the parent cells of 13.3 µM (Fig. 2). In addition, it is also conceivable that *E. coli* minicells could have a compromised sugar efflux (potentially ATP dependent) which could lead to higher cytoplasmic concentrations of sugars such as arabinose^46^.

Obtaining maximum purity of the SimCell suspensions involved a trade-off with the maximum yield and functionality of the cells. It was also noted that using carbenicillin in place of ampicillin throughout the incubation and separation process resulted in negligible SimCell function. This could be caused by carbenicillin maintaining metabolic pressure on the SimCells and thus utilising valuable resources, as opposed to ampicillin which thermally degrades within a short period of time (data not shown). The limitations of these minicell-derived SimCells include the limitation of cell content due to small compartment size, the inability of minicells to repair and replace vital cellular components, and their finite energy resource. In the future these issues may be addressed by incorporating ATP-producing components such as trans-membrane proteorhodopsin in order to generate ATP from sunlight. However, their finite lifespan may also be seen as an advantage in terms of environmental containment. In addition, using SimCells would not overcome the issue of natural competence that allows the uptake of extracellular DNA; another method would be required such as the use of a synthetically designed non-canonical nucleic acid code. This, in combination with the SimCell chassis, could yield a highly genetically confined, non-replicating cell with numerous potential uses. SimCells thus have the potential to circumvent the negative inherent features of fully functional replicating bacteria, can be purified in large quantities, and, as both their cell membrane and genetic components can be modified, they can be considered to be highly modifiable ‘biosensing bioparticles’.

In addition, non-replicating cells such as SimCells have the potential to benefit studies focused on artificial microbial consortia construction. Currently, artificial communities struggle to maintain their designed function for extended periods due to proliferation or cell death disrupting its delicate structure^47^. The use of multiple species of SimCell as opposed to whole cells could enable continued structure, as without proliferation, the spatial and temporal distribution of cells can be maintained. In conclusion, this proof-of-concept work examined three different inducible systems, which were demonstrated to be functional within the SimCell chassis. The detection of low concentrations of intracellular small molecules into a discernible output was made potentially even more sensitive by the compartmentalisation of such molecules into a small contained membrane. We therefore propose the concept of the SimCell as a highly customisable chassis for synthetic biology.

## Materials and Methods

### Chemicals and reagents

All reagents were purchased from Sigma Aldrich (Dorset, UK) unless otherwise stated. Antibiotics were obtained from Fisher Scientific (UK). All inducers were dissolved in sterile 0.01 M phosphate buffered saline (PBS) solution.

### Transmission Electron Microscopy (TEM) imaging of cells

A 5 ml cell suspension was applied onto a 400 mesh carbon coated copper EM grid (Arar Scientific, UK) which was treated by glow discharge for 30 seconds before use. The sample solution was left on the grid for 1 minute. Excess solution was blotted away by touching the grid edge onto the surface of Whatman filter paper. The grid was washed twice with distilled water, stained with 0.75% (w/v) uranyl formate for 30 seconds and dried in air before being imaged. Electron micrographs were recorded with a Philips CM100 microscope equipped with a 1 K × 1 K Gatan Multiscan 794 CCD camera at ×3796 magnification.

### Culture and Purification of SimCells

The strains and plasmids used in this study are shown in Table 2. *E. coli* MC1000 ΔminD cells^15^ were made chemically competent before transformation with high copy-number plasmids pCdaR, pAcuR^31^ and pBAD which all contain the *gfp* gene (Fig. S1).

**Table 2:** Bacterial strain and plasmids used in this study.

Strains	Description	Reference
*E. coli* MC1000 Δ*min*D	MC1000 *minD::kan*	15
Plasmids		
pBAD	*gfp* gene expression is under control of L-arabinose	This study
pAcuR	*gfp* gene expression is under control of acrylate	31
pCdaR	*gfp* gene expression is under control of glucarate	31

The construction of pBAD is described as follows. The *gfp* gene was isolated by PCR from the plasmid pET23b-pduP18-GFP (kind gift from Prof. Martin Warren, University of Kent, UK) using Q5 reaction mix (New England Biolabs) and the following primers: forward 5′-GCTCCATGGGAAACACTTCCAGAACTTGAAACCCTTATTCG-3′ and reverse 5′-GCTAAGCTTAGTGATGGTGATGGTGATGCGATCC-3′. The resulting PCR product contains an extra N-terminal sequence encoding a 22 amino acid tag for targeting to bacterial micro-compartments but this does not affect GFP expression. The *gfp* PCR product was digested with *Nco*I and *Hind*III which generated a 0.2 Kb *NcoI-Hind*III upstream fragment and a 0.6 Kb *Hind*III downstream fragment, as the *gfp* gene carries an internal *Hind*III site. The upstream fragment was ligated into *NcoI-Hind*III cut pBAD/Myc-His C vector (Invitrogen, UK) which was then cut with *Hind*III and the downstream *Hind*III fragment inserted to generate the full-length gene.

Following heat shock, transformants of *E. coli* MC1000 Δ*min*D with plasmids pCdaR, pAcuR and pBAD were separately screened on Luria-Bertani (LB) agar selection plates containing 100 µg/ml ampicillin. A single colony from each plate was cultured at 37 °C with continuous shaking at 120 rpm overnight in 5 ml LB broth supplemented with 100 µg/ml ampicillin (LB amp). Overnight culture was added to fresh LB amp in a ratio of 1:1000 and cultured for 24 hours. For the minicell purification, a modification of a previously described method^48^ was used in order to obtain a high yield and purity, while maintaining maximum ATP within the minicells. Overnight culture was centrifuged at 4 °C at increasing speeds of 1000 g increments, from 1000 g to 4000 g for 10 minutes at each step to remove parent cells from the suspension. The supernatant was subsequently treated with 100 µg/ml ceftriaxone and incubated at 4 °C overnight. Following further centrifugation at 4000 g for 15 minutes to pellet any remaining lysed and elongated parent cells as a result of the ceftriaxone treatment, the supernatant was passed through a 0.22 µm nitrocellulose membrane and the minicells re-suspended from the membrane into sterile PBS solution to concentrate them 100-fold. PBS was chosen as it is a cost-effective medium in which to suspend the cells and to maintain osmotic balance. Minicell samples were maintained at 4 °C prior to testing. The purity of minicell suspensions was determined by a plate-count method after 24 h incubation on LB-amp agar plates at 37 °C and conducted in triplicate.

### GFP fluorescence measurements

Stock solutions of L-arabinose, glucarate (D-saccharic acid, potassium salt) and acrylate (acrylic acid, sodium salt) were made in deionised water and sterilised using a 0.2 µm syringe filter to be tested with cells containing pBAD, pCdaR and pAcuR respectively. One hundred µL of purified minicells was combined with 100 µL of inducing agent solution in a 96 well black sided, clear-bottomed plate (Nunclon, UK) in triplicate. The plate was then placed into a BioTek Synergy HT microplate reader (BioTek Corporation, UK) maintained at 37 °C with continuous shaking with simultaneous reads of fluorescence (excitation: 480 nm, emission: 520 nm) and optical density (OD) at 600 nm taken.

### Quantification of ATP

ATP was quantified using the Promega BacTiterGlo ^TM^ (Promega, Madison, WI, USA) assay with 100 µL reagent plus 100 µL *E. coli* MC1000 with pCdaR cells suspended in sterile PBS. A concentration range of 1 pM to 1 µM of dATP was used to construct a calibration curve (R^2^ = 0.99). An aliquot of parent cell culture in stationary phase was taken prior to purification and adjusted to the same OD as purified SimCells in PBS prior to testing for intracellular ATP. The same batch of purified pCdaR SimCells were maintained at either 4 °C or 37 °C for 24 h after purification before testing in triplicate.

### Visualisation of SimCells

Fluorescence of SimCells was visualised using a Motica BA210 digital microscope with Moticam 580INT display output after 24 hours of induction. Ten µl of each concentration was taken from the well and placed on a glass slide with coverslip. Images were taken at 40× magnification. Light micrographs were subsequently analysed using ImageJ version 1.50b, with fluorescent maxima automatically counted after consistent MaxEntropy thresholding, using the method as previously described by Siritantikorn and co-workers^49^. Four images were processed for each concentration of inducing agent. Statistics were calculated using a T-test, with a p value of below 0.05 determining significance at a 95% confidence level.

## Electronic supplementary material

Supplementary Information
