# Supplementary material for: Development of SimCells as a novel chassis for functional biosensors
Source: Sci Rep. 2017 Aug 3;7:7261. doi: 10.1038/s41598-017-07391-6 (PMC5543166; doi:10.1038/s41598-017-07391-6)
Supplement: Supplementary file 1 — Supplementary Information [file 41598_2017_7391_MOESM1_ESM.pdf]

Supplementary information

Development of SimCells as a novel chassis for functional biosensors

Cordelia P. N. Rampley[1], Paul A. Davison[2], Pu Qian[2], Gail M. Preston[3], C. Neil Hunter[2], Ian P. Thompson[1], Ling Juan Wu[4] and Wei E. Huang[1]

1. Department of Engineering Science, University of Oxford, Parks Road, Oxford, OX1 3PJ, United Kingdom.
2. Department of Molecular Biology and Biotechnology, University of Sheffield, Sheffield, S10 2TN, United Kingdom.
3. Department of Plant Sciences, University of Oxford, South Parks Road, Oxford OX1 3RB, Oxford, United Kingdom.
4. The Centre for Bacterial Cell Biology, Institute for Cell and Molecular Biosciences, Newcastle University, Richardson Road, Newcastle upon Tyne, NE2 4AX, United Kingdom

*Corresponding author: Wei E. Huang

wei.huang@eng.ox.ac.uk

Telephone: +44 (0)1865 283786, Fax: +44 (0)1865 374992

Mathematical Theory

Inducer and regulation protein binding

An inducer molecule S binds regulation protein R and activates it. Given that the total regulatory protein level is R_T, n molecules of S_0 (initial concentration of inducer) bind R to become R^*, whilst the unbound is R_u. According to the conservation law,

$R^*+R_u=R_T$ (1)

Now consider two processes: R^* formation and R^* dissociation.

In R^* formation, R^* is formed due to collision of R and n molecules of S_0 (if S_0 is not degraded, it will be constant),

The collision rate = $k_{on}R_uS_0{}^n$ (2)

where k_{on} is the binding constant of substrate S to R.

R^* dissociation: R^* dissociates from inducer molecule S to form R,

where the dissociation rate = $k_{off}R^*$ (3)

and k_{off} is the dissociation constant of substrate S to R.

The net change of R^* can be described as

$$\frac{dR^*}{dt} = k_{on}R_uS_0^n - k_{off}R^* \qquad (4)$$

When the equation is solved, R^* at time t is,

$$R^*(t) = R_{st}^*\left(1 - e^{-\left(k_{on}S_0^n + k_{off}\right)t}\right) \qquad (5)$$

where $R_{st}{}^*$ is at the steady state,

$$R_{st}^* = \frac{R_TS_0^n}{S_0^n + K_x^n} \qquad (6)$$

$$K_x^n = \frac{k_{off}}{k_{on}} \qquad (7)$$

K_x is the dissociation constant and n is the Hill coefficient; n is 1~6.

In many cases, the binding of molecule S and regulation protein R can reach equilibrium within milliseconds, so $R^*=R_{st}{}^*$.

Input function of regulated gene (activator)

In the case of activation, gene expression in SimCells or cell induction in PBS,

activation promoter activity = $\frac{\beta_m R^*}{R^*+K_d}$ (8)

Since there are limited resources to express proteins in SimCells and cell induction in PBS, we add a term to slow down the promoter activity. Protein (e.g. GFP) production rate can be described as,

$$\frac{dP}{dt} = \frac{\beta_m R^*}{R^*+K_d} - \alpha_d P \quad (9)$$

where α_d= decay constant of protein production, due to the depletion of resources and energy in PBS and SimCells.

$$P(t) = P_{st}(1 - e^{-\alpha_d t}) \quad (10)$$

$$P_{st} = \frac{\beta_m R^*}{\alpha_d (R^*+K_d)} \quad (11)$$

P: GFP unit, β_m , is the maximal transcriptional rate, usually in the range of 10^{-4}~1 unit/s [1]; it is a combined parameter that includes the copy number of the gene circuit, physiological states, and the ratio between the number of GFP proteins and reading unit. Parameters used in the simulation of arabinose induction are summarised in Table S1.

Table S1: Parameters used in simulation of arabinose induction

Parameters	Value	Note and Reference
**Intracellular concentration of regulatory protein**	5×10^{-7} M	500 proteins per cell, given 1/cell volume=1 nM [1]
**Inducer (arabinose) and regulation protein (araC) dissociation constant K_x for interaction between inducer (arabinose) and regulatory protein (araC)**	4×10^{-4} M	[2]
**Hill coefficient for inducer and regulation protein**	1.3	araC is homodimer [3]
**Regulation protein and promoter dissociation constant K_d**	3×10^{-10} M	[4]
**Degradation and dilution constant α**	1.73×10^{-2} (min^{-1})	Doubling time $t_{1/2}$ = 40 min, $\alpha=\ln2/t_{1/2}$
**Production decay rate due to running out of fuel in SimCells α_d**	5 ~ 8.33×10^{-3} (min^{-1})	Arabinose can be used as an energy source, so the higher the concentration of arabinose, the lower the decay rate
**Maximal transcriptional rate βm in SimCells**	14 (GFP unit min^{-1})*	The range of transcription rate is 6×10^{-3} -60 mRNA/min [1]
**Measurement baseline in SimCells**	8400 (GFP unit)	
**Production decay rate due to running out of fuel in Cells in PBS α_d**	1.25 ~ 1.67×10^{-3} (min^{-1})	Arabinose can be used as energy source, so the higher the concentration of arabinose, the lower the decay rate. The decay rate is smaller as cells in PBS are larger than SimCells
**Maximal transcriptional rate βm in cells in PBS**	42 (GFP unit min^{-1})*	The range of transcription rate is 6×10^{-3} -60 mRNA/min [1]
**Measurement baseline in cells in PBS**	4400 (GFP unit)	

Note: *GFP unit is instrument reading of GFP not, protein numbers.

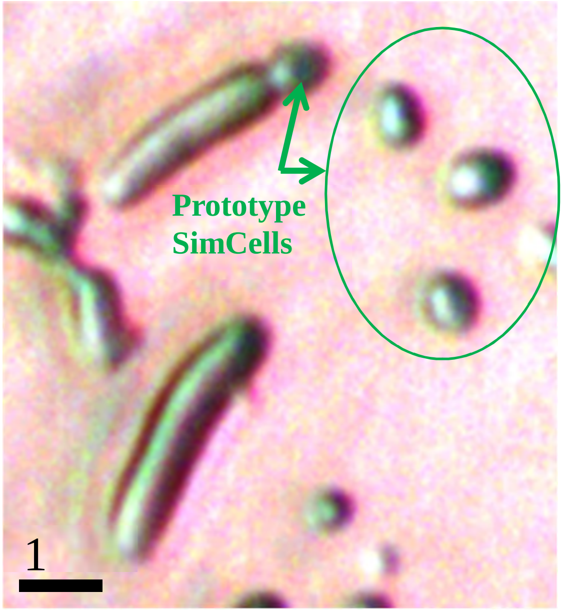

(A)

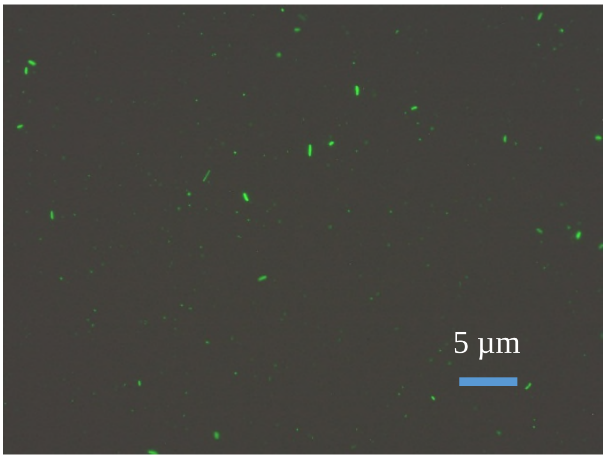

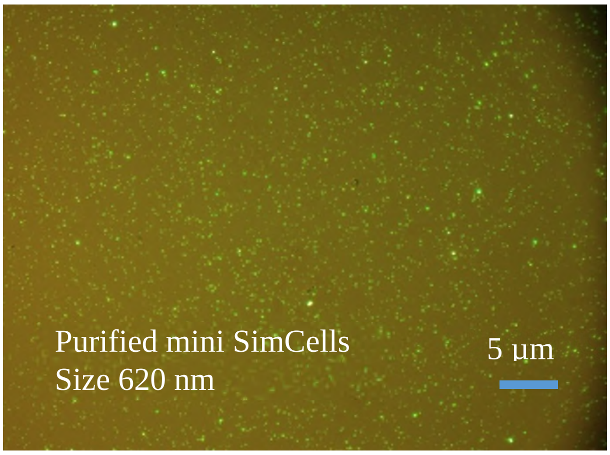

(B) **(C)**

Figure S1. *E. coli* MC1000 parent cell and minicell. (A) A snapshot of parent cells producing SimCells. (B) unpurified and induced pCdaR SimCells with parent cells (C) Uniformity of purified SimCells stained by SYTO 9 green fluorescent nucleic acid stain.

A

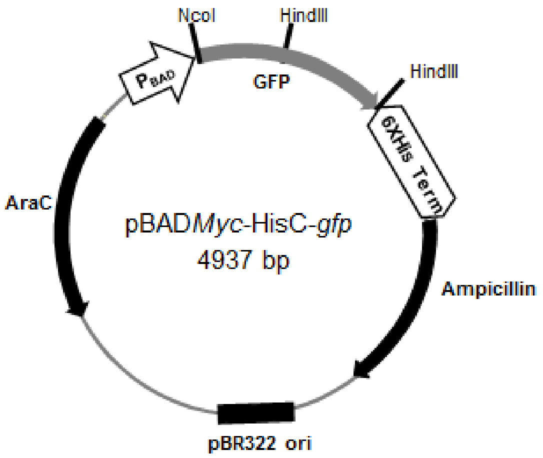

B

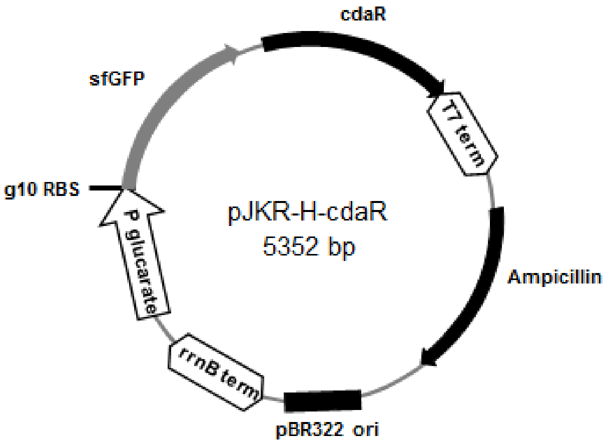

C

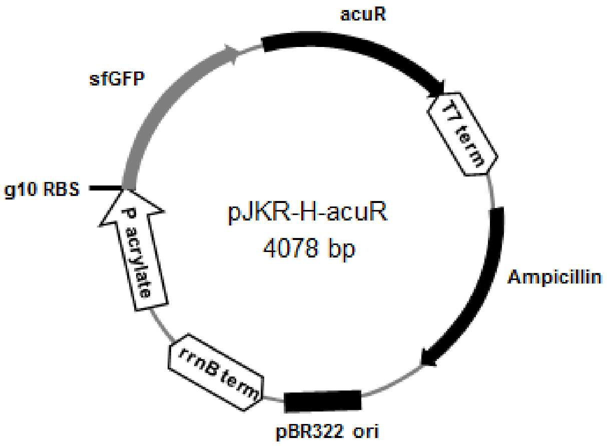

Figure S2: Plasmid maps of A) pBAD, B) pCdaR and C) pAcuR

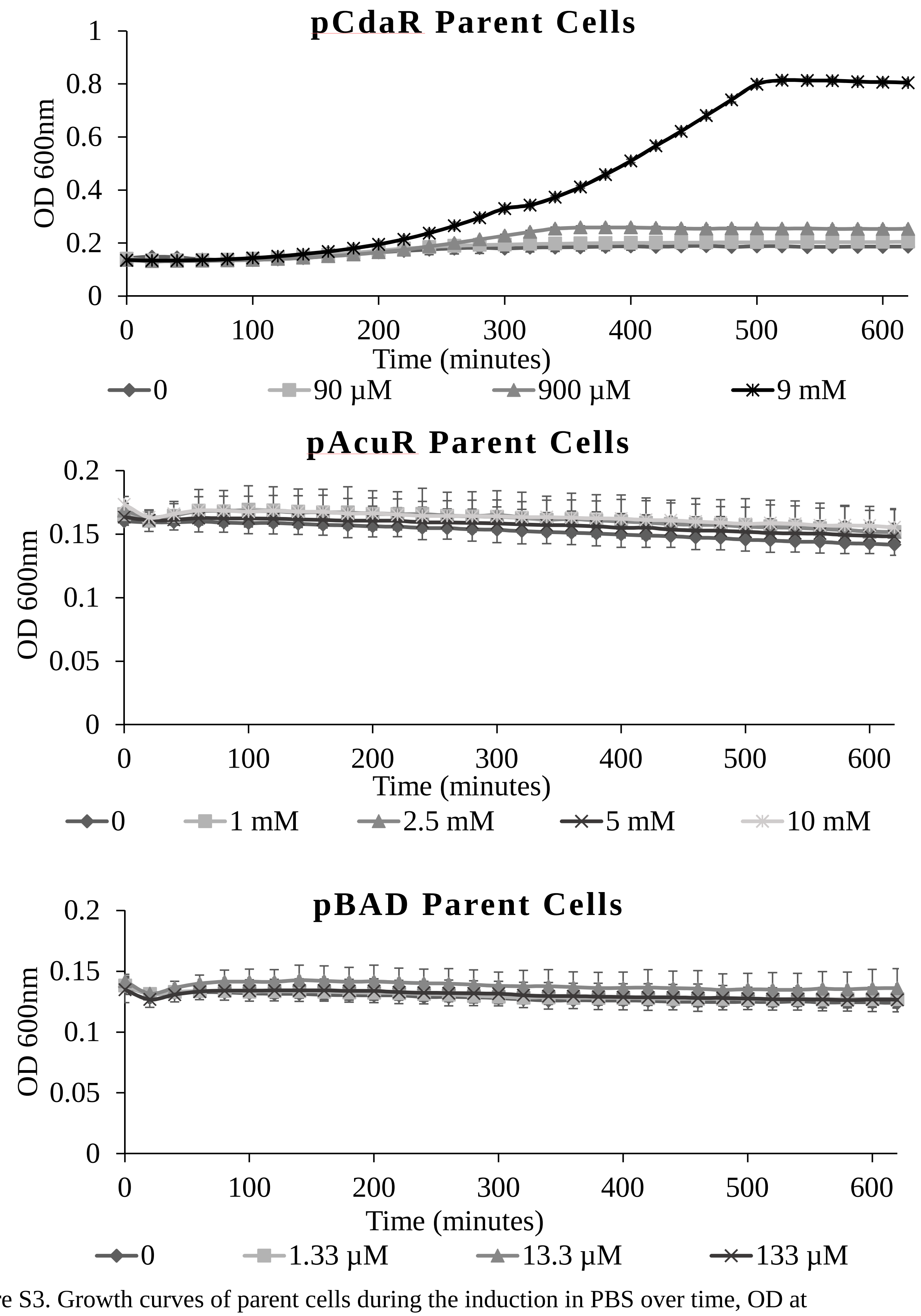

Figure S3. Growth curves of parent cells during the induction in PBS over time, OD at 600nm (n=4). Error bars denote one standard deviation above and below the mean. (A) pCdaR induced by glucarate. (B) pAcuR induced by acrylate. (C) pBAD induced by arabinose.

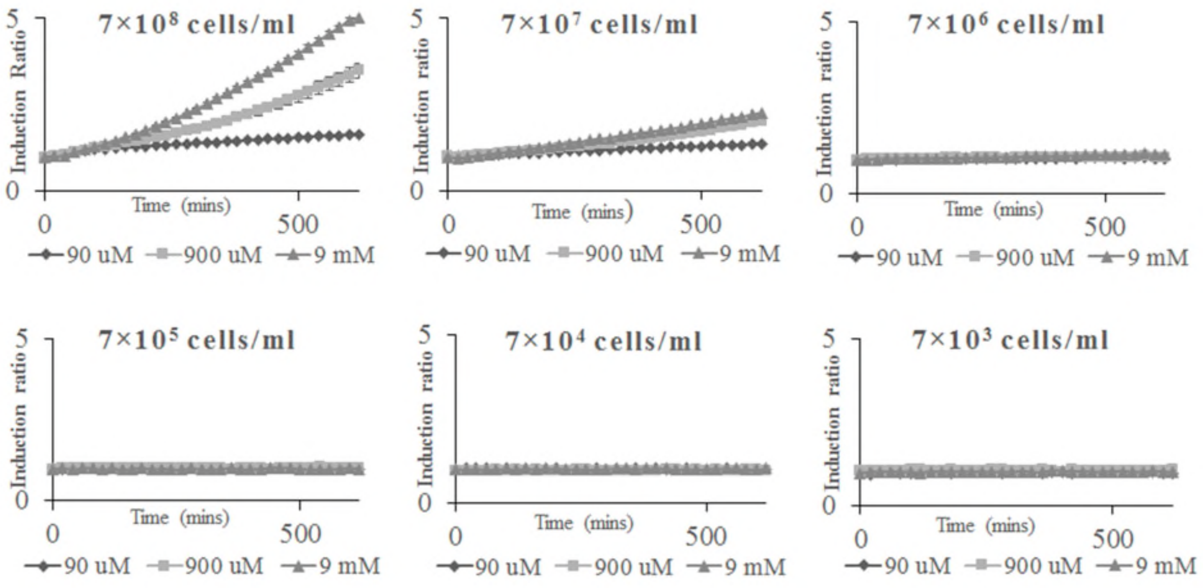

Figure S4. Induction of pCdaR parent cells in PBS over time, as determined by GFP fluorescence per unit OD at 600nm of cells in serial dilution (n=3). Cells quantified by plate count method (n=3). Induction ratio of induced cells was significantly different from the control ($p<0.05$) until dilution reached 7×10^7 cells/ml. Cell population less than 7×10^6 cells/ml has no detectable GFP activation. Error bars denote one standard deviation above and below the mean.

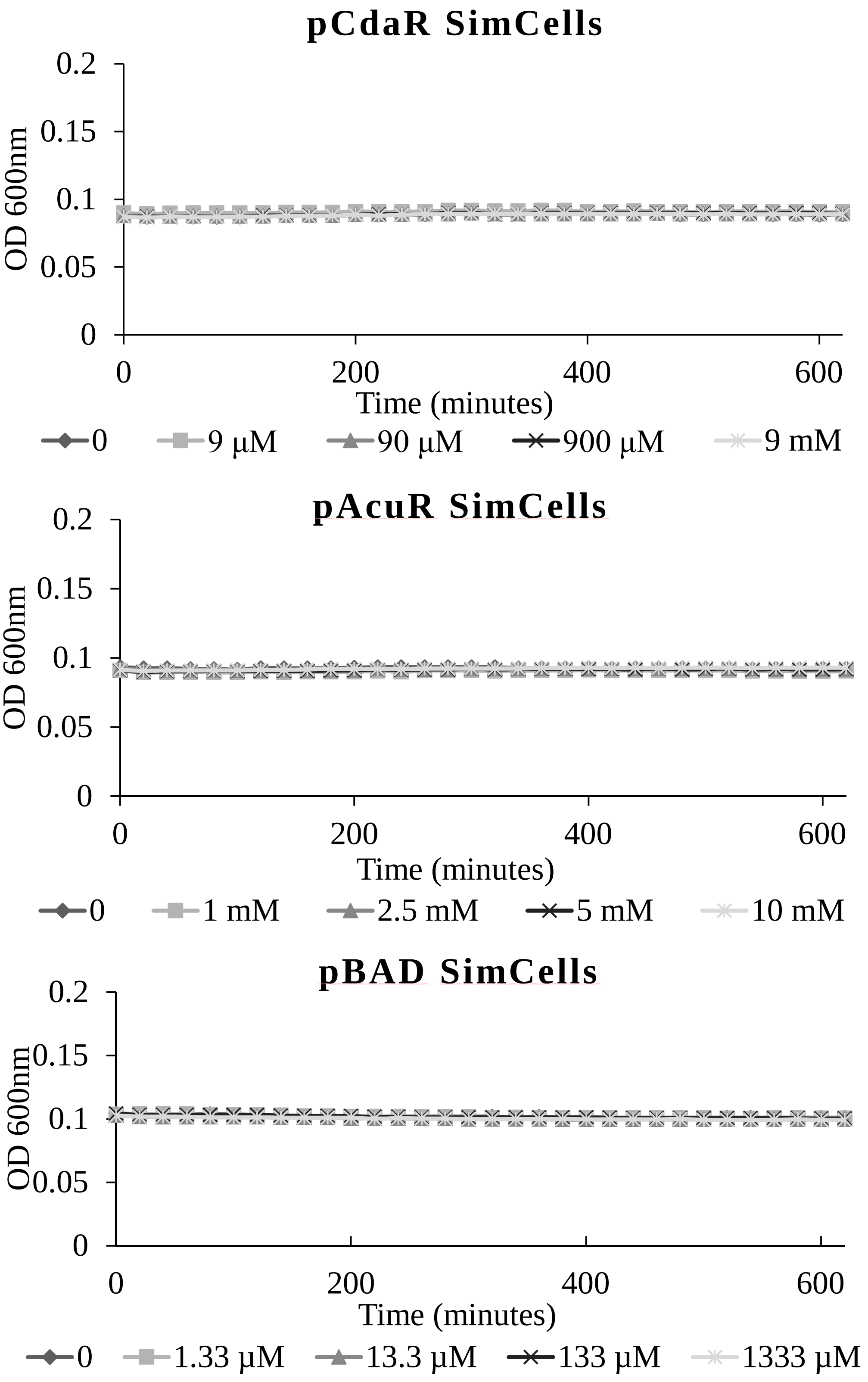

Figure S5. Growth curves of purified SimCells during induction in PBS over time as measured by OD at 600nm (n=4). Error bars denote one standard deviation above and below the mean. Graphs show (A) pCdaR induced by glucarate. (B) pAcuR induced by acrylate. (C) pBAD induced by arabinose.

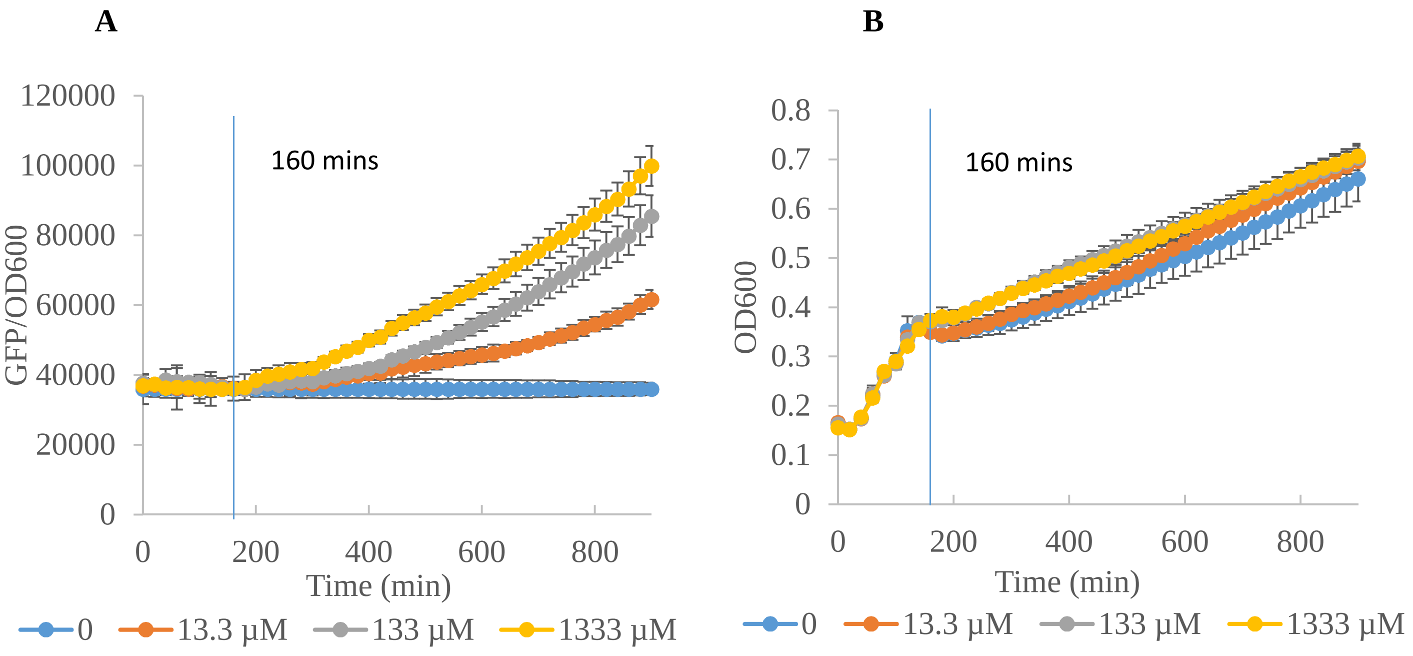

Figure S6. The delayed response of arabinose induction in *E. coli* MC1000 with pBAD plasmid was related to cell growth in LB as shown by (A) the induction response curve over time and (B) the OD 600nm growth curve. Error bars represent one standard deviation above and below the mean (n=4). Line at 160 minutes represents the point at which the initial rapid rate of cell proliferation decreases, which corresponds with the increase in GPF production.

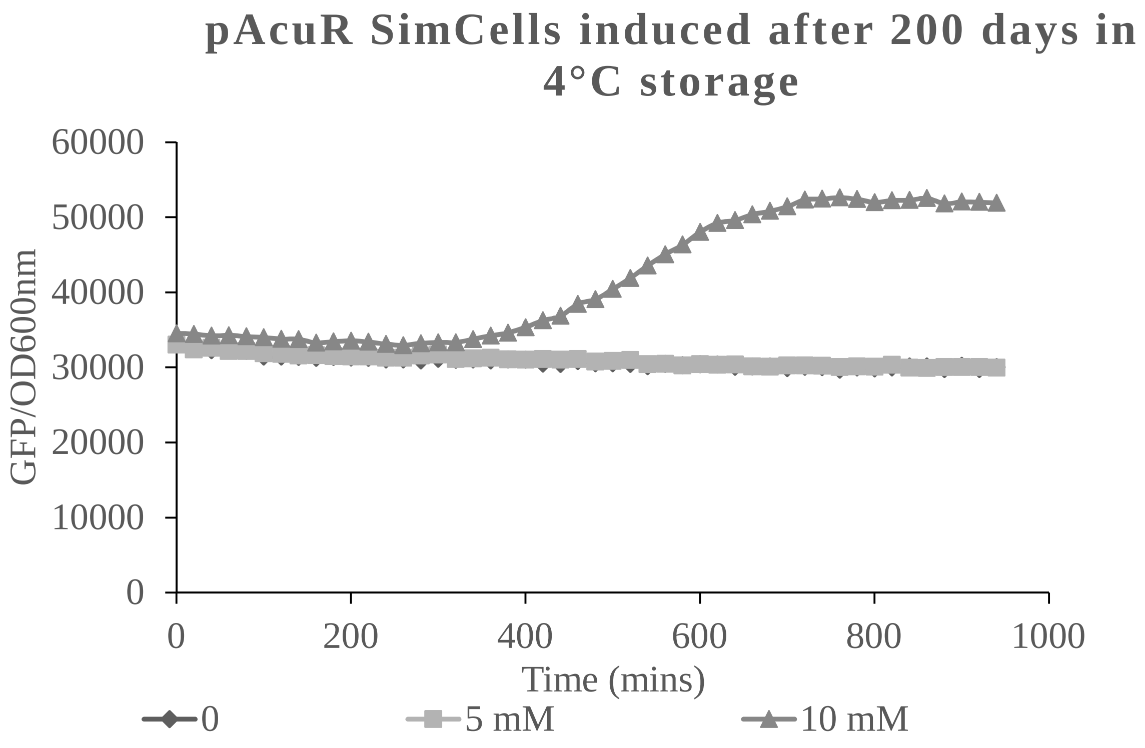

Figure S7. Induction of 200-day-old pAcuR SimCells by acrylate in PBS over time, as determined by GFP fluorescence per unit OD at 600nm. Samples were stored at 4 ºC in centrifuge tubes.

References:

1 Alon, U. *Introduction to system biology: design principles of biological circuits*. (Taylor & Francis Group LLC, 2007).

2 Schleif, R. AraC protein, regulation of the l-arabinose operon in Escherichia coli, and the light switch mechanism of AraC action. *Fems Microbiology Reviews* **34**, 779-796, doi:10.1111/j.1574-6976.2010.00226.x (2010).

3 Schleif, R. Regulation of the L-arabinose operon of Escherichia coli. *Trends in Genetics* **16**, 559-565, doi:10.1016/s0168-9525(00)02153-3 (2000).

4 Zhang, X., Reeder, T. & Schleif, R. Transcription activation parameters at ara p(BAD). *Journal of Molecular Biology* **258**, 14-24, doi:10.1006/jmbi.1996.0230 (1996).
